# KAN-DeScoD: Kolmogorov–Arnold Network Enhanced Deep Score-Based Diffusion Model for ECG Denoising

**DOI:** 10.3390/s26072213

**Published:** 2026-04-03

**Authors:** Zhixin Shu, Deqiu Zhai, Lei Huang, Ying Zhang, Tao Liu

**Affiliations:** 1 School of Artificial Intelligence, Nanjing University of Information Science and Technology, Nanjing 210044, China; zhixin.shu@nuist.edu.cn (Z.S.); deqiu.zhai@nuist.edu.cn (D.Z.); lei.huang@nuist.edu.cn (L.H.); 2 Department of Medical Physics and Biomedical Engineering, University of London College, London WC1E 6BT, UK; ying.zhang.18@ucl.ac.uk; 3 Key Laboratory of Pattern Recognition and Intelligent Information Processing, Institutions of Higher Education of Sichuan Province, Chengdu University, Chengdu 610106, China

**Keywords:** deep score-based diffusion model, ECG denoising, Kolmogorov–Arnold network, adaptive activation functions, robustness in high-noise environments

## Abstract

**Highlights:**

**What are the main findings?**

- This paper indicates that the deep score-based diffusion (DeScoD) model lacks flexibility in fitting non-stationary noise and signal morphology changes due to the use of linear transformations in its feature affine transformation layer.
- To address this issue, this paper proposes, for the first time, a Kolmogorov–Arnold network enhanced deep score-based diffusion (KAN-DeScoD) model. This model replaces linear layers with KAN layers and introduces activation sparsity and entropy regularization strategies specifically designed for KAN, significantly enhancing the model’s feature representation and nonlinear approximation capabilities.

**What are the implications of the main findings?**

- Systematic validation on the QT Database and MIT-BIH NSTDB demonstrates that the KAN-DeScoD model outperforms baseline models across multiple key metrics under various noise intensities, enhancing the model’s robustness in high-noise environments and the accuracy of signal reconstruction.
- This paper provides a new perspective in the field of ECG denoising, demonstrating the effectiveness of combining KAN, which has strong theoretical approximation capabilities, with generative diffusion models.

**Abstract:**

Thedeep score-based diffusion (DeScoD) model performs well in electrocardiogram (ECG) denoising tasks. However, due to the theoretical error lower bound in approximating functions with linear transformations, it often lacks flexibility when fitting non-stationary noise, baseline wander, or morphologically variable features such as QRS complexes in ECG signals. In this paper, we propose a Kolmogorov–Arnold network enhanced deep score-based diffusion (KAN-DeScoD) model, which is the first to integrate Kolmogorov–Arnold network (KAN) layers into an ECG denoising diffusion model. By leveraging KAN’s adaptive activation functions, which more finely capture the complex structures within ECG signals, the model’s robustness in high-noise environments, as well as the accuracy and stability of signal reconstruction, are improved. We validate the effectiveness of the proposed method on the QT Database and the MIT-BIH Noise Stress Test Database (NSTDB). Experimental results show that under different shots and noise intensities, ours outperforms the DeScoD model across multiple metrics. The research results demonstrate the effectiveness of introducing KAN, which improves the model’s robustness in high-noise environments and the accuracy of signal reconstruction.

## 1. Introduction

The electrocardiogram (ECG) is an indispensable non-invasive tool for the clinical diagnosis of heart diseases. However, the acquisition process is often contaminated by baseline wander, electromyographic (EMG) interference, and powerline noise. These noises severely obscure key waveforms that reflect cardiac pathological features, such as the P wave, QRS complex, and T wave, thereby increasing the risk of clinical misdiagnosis. In recent years, with the advancement of bioelectronics, new technologies like self-locking conductive cardiac patches for integrated infarcted myocardium have emerged [1], placing higher demands on the real-time acquisition and precise reconstruction of high-quality ECG signals.

Traditional ECG denoising methods mainly rely on finite impulse response filters [2,3] and infinite impulse response filters [4,5]. Although these methods are simple and efficient in suppressing linear noise within specific frequency bands [6], they are limited in handling non-stationary noise that significantly overlaps with the ECG signal spectrum, such as complex EMG interference. Additionally, conventional adaptive filtering algorithms often struggle to balance denoising strength and waveform preservation when dealing with non-Gaussian interferences like alpha-stable noise [7].

With the rapid development of artificial intelligence technology, deep learning has been widely applied to various biological signal processing tasks. Research shows that neural networks have significant advantages in handling complex nonlinear bioelectrical signals, such as sEMG-based motion recognition [8], EEG-based driver fatigue detection [9], the diagnosis of sarcopenia or muscle fatigue in elderly populations [10], and fast denoising filtering in ultrasound localization microscopy imaging [11]. In the specific domain of signal denoising, several architectural paradigms have emerged:

1. Denoising Autoencoders: Early deep learning attempts utilized Denoising Autoencoders [12,13] and Fully Convolutional Denoising Autoencoders [14] to learn robust feature representations by reconstructing clean signals from corrupted inputs. While they reduced the dependence on prior noise models, they often struggled with high-dimensional data and local feature preservation.
2. Deep Recurrent Neural Networks: To account for the inherent temporal dependencies in ECG data, Deep Recurrent Neural Networks [15] and Long short-term memory [16,17] were introduced. These models excel at capturing long-term sequential patterns but often face challenges such as vanishing gradients and high computational latency during inference.
3. Generative adversarial networks (GANs): Generative approaches, such as Conditional GANs [18], have injected new vitality into the field by learning the distribution of clean ECG data through adversarial training. Recent innovations, including GANs with pyramid coordinate attention [19] or Gated Swin Transformers [20], have further enhanced image and signal restoration. However, GANs are notoriously difficult to train and can occasionally introduce “hallucinated” artifacts that compromise clinical reliability.

Beyond these, advanced frameworks like Auto-weighted Feature Extractors [21], Broad Vision Transformers [22], and Graph Convolutional Networks [23] have pushed the boundaries of feature extraction in DNA methylation and point cloud assessment. Learning-driven frameworks have also shown robustness in latency compensation for teleoperation [24], channel-wise attention for steganography [25], and 3D reconstruction from sparse X-ray data [26].

In recent years, score-based diffusion models [27,28] have demonstrated superior generative quality and training stability compared to GANs in fields such as image generation [29] and audio synthesis [30,31]. Innovatively, Li et al. [32] introduced score-based diffusion models into the ECG denoising task. By conditioning on the noisy signal and iteratively generating high-quality reconstructed signals from random Gaussian noise, they achieved a more precise approximation of the real data distribution and exhibited exceptional robustness and stability under extreme noise conditions.

Although the deep score-based diffusion (DeScoD) model has achieved significant results in ECG denoising [32], its linear transformations within the feature affine transformation method have limitations in expressive power when modeling the mapping relationship from complex noise to clean signals [33,34,35]. The combination of standard linear transformations with fixed activation functions may fail to adequately capture the unique spatiotemporal features and noise structures of ECG signals, resulting in denoised signals that exhibit waveform smoothing or loss of detail [36]. This is particularly problematic given that even high-fidelity representation methods, such as those utilizing improved ensemble empirical mode decomposition for data compression [37], rely on the preservation of fine-grained signal components that linear layers often suppress. This can affect physicians’ accurate assessment of critical diagnostic features. Additionally, when dealing with non-stationary noise and sudden interference, the fixed response characteristics of linear transformations lead to unstable denoising performance [38]. They struggle to dynamically adjust their response to different noise patterns, which can cause residual noise or excessive signal smoothing. This instability reduces the reliability of diagnostic outcomes and may obscure transient arrhythmias and other key clinical events, especially during long-term monitoring.

Kolmogorov–Arnold network (KAN), with its theoretical universal approximation capability and adaptive nonlinear characteristics [36], has demonstrated its superiority and robustness in multiple fields [39,40,41] such as ECG classification [42,43,44]. This provides a potential solution to the problem that linear transformations cannot adequately capture the unique spatiotemporal features and noise structure of ECG signals. Combining KAN’s flexible function approximation ability with the generative advantages of diffusion models holds promise for further enhancing the accuracy and robustness of ECG denoising.

Motivated by the exceptional function approximation capabilities of KAN, we propose the Kolmogorov–Arnold network enhanced deep score-based diffusion (KAN-DeScoD) model, which is the first to integrate KAN layers into an ECG denoising diffusion model. By replacing fixed activation functions with learnable univariate functions (B-splines), ours offers a more flexible mathematical framework for capturing transient changes and nonlinear structures in ECG signals. The main contributions of this paper are summarized as follows:

1. Firstly, we are the first to integrate KAN layers into an ECG denoising diffusion model. We design the KAN-DeScoD model by replacing the linear transformation layers in the original model’s feature affine transformation layers with KAN layers, thereby enhancing feature representation capability.
2. Secondly, we propose activation sparsity and entropy regularization strategies tailored for KAN.
3. Finally, extensive evaluations on the QT Database and MIT-BIH Noise Stress Test Database (NSTDB) demonstrate that ours significantly outperforms baseline models in signal fidelity and R-peak preservation, especially in high-noise environments.

The remainder of this paper is organized as follows. Section 2 briefly introduces the related work of this paper, such as the DeScoD model, the feature affine transformation method, and the KAN model. Section 3 presents the improved method proposed in this paper. Section 4 covers the database description, performance metrics, and experimental configuration. Section 5 provides the experimental results. Section 6 discusses the experimental results. Section 7 analyzes the limitations of the model and future work. Finally, Section 8 concludes this paper.

## 2. Related Works

### 2.1. DeScoD Model

**Figure 1:**
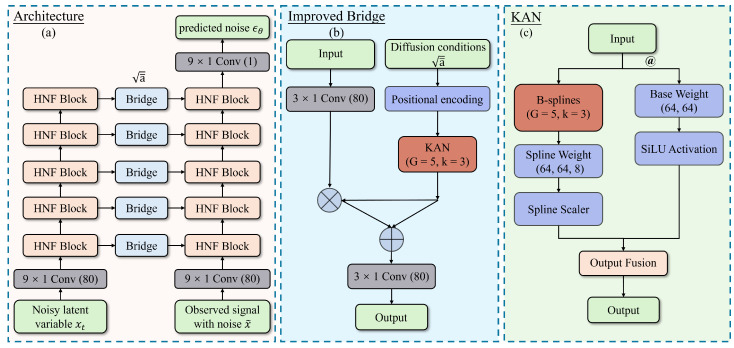
This neural network architecture adopts an improved diffusion model design. The overall network consists of multiple layers of HNF Blocks, with features at different levels connected through Bridge modules (**a**). We specifically create an improved Bridge module by replacing the 1 × 1 convolution layer with a KAN layer (**b**). The Kolmogorov–Arnold network (KAN) layer uses a dual-path structure, which is the basic path and the spline path, and the outputs of the two paths are summed at the end (**c**).

The DeScoD model [32] is a diffusion model-based denoising framework designed for ECG signals (Figure 1a). Its core idea is to utilize a conditional generation approach, simulating the physical process of gradual noise addition and reverse removal to achieve high-quality signal reconstruction [27]. Unlike traditional unconditional diffusion models, the main innovation of this model lies in introducing the noisy ECG signal as a conditional guide [45]. This enables the denoising process to adaptively handle specific noise patterns (such as baseline wander, EMG interference, and powerline noise), thereby effectively suppressing noise while preserving important clinical waveform features.

The forward diffusion process can be formulated as a Markov chain that gradually transforms the clean ECG signal $x_{0}$ into an approximately isotropic Gaussian noise distribution $x_{T}$ over *T* steps:

(1) $$q\left(x_{t}|x_{t-1}\right)=N\left(x_{t};\sqrt{1-\beta_{t}}x_{t-1},\beta_{t}I\right)$$

where $x_{t}$ denotes the latent variable at timestep *t*; $\beta_{t}$ represents the predefined noise schedule parameter; I denotes the identity matrix.

The reverse denoising process is a step-by-step generation process guided by a trained conditional denoising network. Starting from the pure noise $x_{T}$ and leveraging the noisy ECG observation $\tilde{x}$ as a conditional guide, the model progressively recovers the clean signal $x_{0}$:

(2) $$p_{\theta }\left(x_{t-1}|x_{t},\tilde{x}\right)=N\left(x_{t-1};\mu_{\theta }\left(x_{t},t,\tilde{x}\right),\sigma_{t}^{2}I\right)$$

where $\tilde{x}$ denotes the noisy ECG signal used as the conditional input. This conditional guidance enables the denoising process to adaptively handle specific noise patterns while preserving critical clinical morphological features.

### 2.2. Feature Affine Transformation Method

In the DeScoD model, feature affine transformation is primarily implemented via the feature affine transformation layers within the Bridge blocks. This layer is an improvement upon Feature-wise Linear Modulation [46], used to effectively fuse noise level information (i.e., the diffusion step) with input features during the denoising process of the diffusion model. However, ECG signals possess complex nonlinear characteristics such as the morphological diversity of QRS complexes. Simple linear mappings struggle to fully capture these detailed structures and dynamic variations within the signal, leading to insufficient fusion of conditional information, which in turn affects both denoising effectiveness and waveform fidelity. Therefore, nonlinear feature extraction is necessary [47].

### 2.3. KAN Model

The theoretical foundation of KAN stems from the Kolmogorov–Arnold representation theorem, which states that any multivariate continuous function can be expressed as a finite composition of univariate functions [36]. The specific mathematical expression is as follows:

For any continuous function $f:{\left[0,\;1\right]}^{n}\to R$, there exist univariate functions $\phi_{q,p}$ and $\psi_{q}$:

(3) $$f\left(x_{1},\ldots ,x_{n}\right)=\sum_{q=1}^{2n+1}\psi_{q}\left(\sum_{p=1}^{n}\phi_{q,p}\left(x_{p}\right)\right)$$

where both $\psi_{q}$ and $\phi_{q,p}$ are univariate functions.

**Figure 2:**
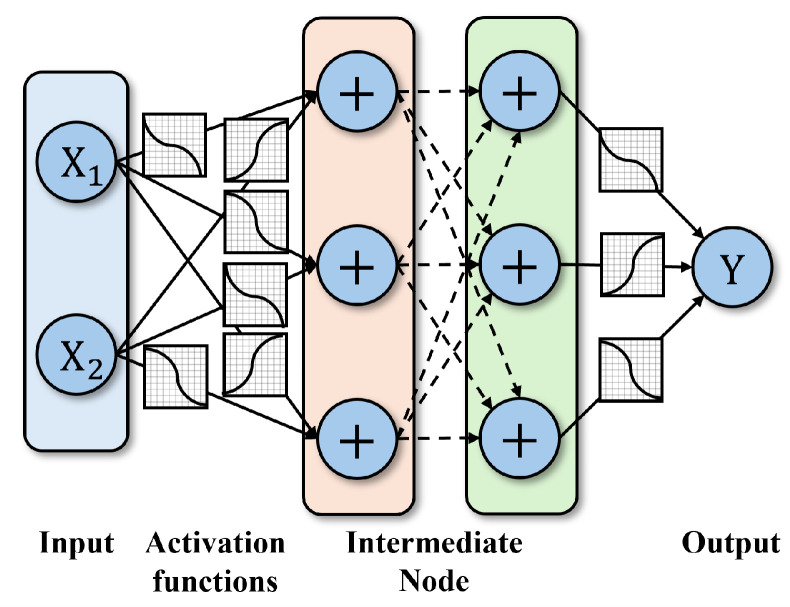
The architecture of the KAN model.

The core characteristic of the KAN model lies in replacing the combination of fixed activation functions and linear weights found in traditional neural networks with learnable univariate functions. As shown in Figure 2, each layer of a KAN consists of multiple learnable nonlinear functions, which are typically parameterized using B-splines or other smooth function bases. This structure endows KAN with stronger expressive power and interpretability, enabling it to achieve complex nonlinear mappings with fewer parameters. KAN has demonstrated superior performance compared to traditional multi-layer perceptrons (MLP) in various scientific computing and data fitting tasks [36]. In ECG signal processing tasks, KAN holds the promise of more finely modeling the local morphological features and global rhythmic patterns within signals, thereby providing more powerful nonlinear modeling capabilities for conditional information fusion and signal reconstruction.

## 3. Methodology

### 3.1. Feature Affine Transformation Layer

This paper improves the linear transformation layer of the characteristic affine transformation layer of the DeScoD model. Traditional linear transformation has a limited Vapnik–Chervonenkis dimension, which shows insufficient learning ability for complex noise patterns in ECG signals, especially when dealing with non-stationary noise and transient interference. The superior function approximation capability of the KAN makes it more suitable for this task. Therefore, we replace the linear layer in the feature affine transformation layer with a KAN layer (Figure 1b).

In the original architecture, the feature affine transformation employs a standard linear transformation framework, comprising a positional encoding layer (4), a linear transformation layer (5), and the feature affine transformation (6):

(4) $$\begin{array}{cc} z & =\mathrm{PE}\left(\sigma_{t}\right)=\left[\sin\left(\sigma_{t}e^{-0\lambda }\right),\cos\left(\sigma_{t}e^{-0\lambda }\right),\;\ldots \;,\sin\left(\sigma_{t}e^{-(d/2-1)\lambda }\right),\cos\left(\sigma_{t}e^{-(d/2-1)\lambda }\right)\right] \end{array}$$

(5) $$\gamma_{1},\beta_{1}=W\cdot z+b$$

(6) $$h_{\mathrm{out}}=\left(1+\gamma_{1}\right)⊙h_{\mathrm{in}}+\beta_{1}$$

where $\lambda =\frac{\ln(10,000)}{\left(d/2\right)}$; z denotes the input feature vector; ⊙ denotes element-wise multiplication.

The KAN transform layer (7) replaces the linear layer, which is composed of the basis function part (8) and the spline function part (9), and the following corresponding outputs (10):

(7) $$\gamma_{2},\beta_{2}=\mathrm{KAN}\left(z\right)=y_{\mathrm{base}}+y_{\mathrm{spline}}$$

(8) $$y_{\mathrm{base}}=W_{\mathrm{base}}\cdot \sigma \left(z\right)$$

(9) $$y_{\mathrm{spline}}=W_{\mathrm{spline}}\cdot B\left(z\right)$$

(10) $$h_{\mathrm{out}}=\left(1+\gamma_{2}\right)⊙h_{\mathrm{in}}+\beta_{2}$$

where W denotes a learnable weight matrix; σ(·) denotes an activation function; B(z) denotes the B-spline basis function matrix.

**Figure 3:**
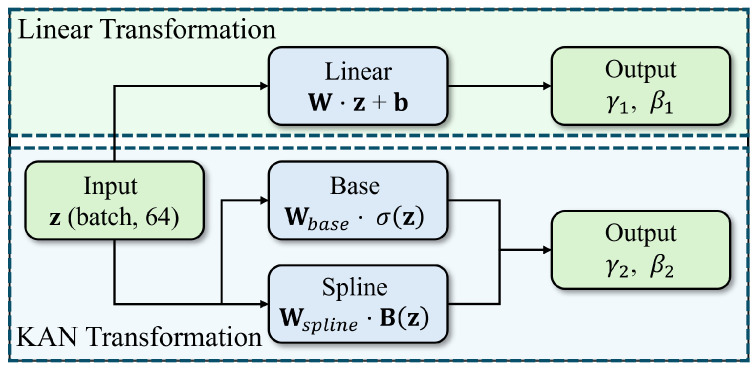
Comparison diagram of linear transformation and KAN transformation.

Figure 3 is a schematic diagram of the linear transformation layer and the KAN transformation layer. The B-spline basis functions can be defined as follows:

(11) $$B_{i,0}\left(z\right)=\left\{\begin{array}{cc} 1 & \mathrm{if}\;s_{i}\leq z<s_{i+1}\\ 0 & \mathrm{otherwise} \end{array}\right.$$

(12) $$B_{i,k}\left(z\right)=\frac{z-s_{i}}{s_{i+k}-s_{i}}B_{i,k-1}\left(z\right)+\frac{s_{i+k+1}-z}{s_{i+k+1}-s_{i+1}}B_{i+1,k-1}\left(z\right)$$

where $B_{i,k}\left(z\right)$ denotes the *i*-th B-spline basis function of order *k*; *k* denotes the B-spline order; $s_{i}$ denotes the *i*-th grid point, defining the support interval of the B-spline basis functions; *i* denotes the index of the grid point, i=0, 1,…,G+k (*G* is the number of grid points, and *k* is the spline order).

**Figure 4:**
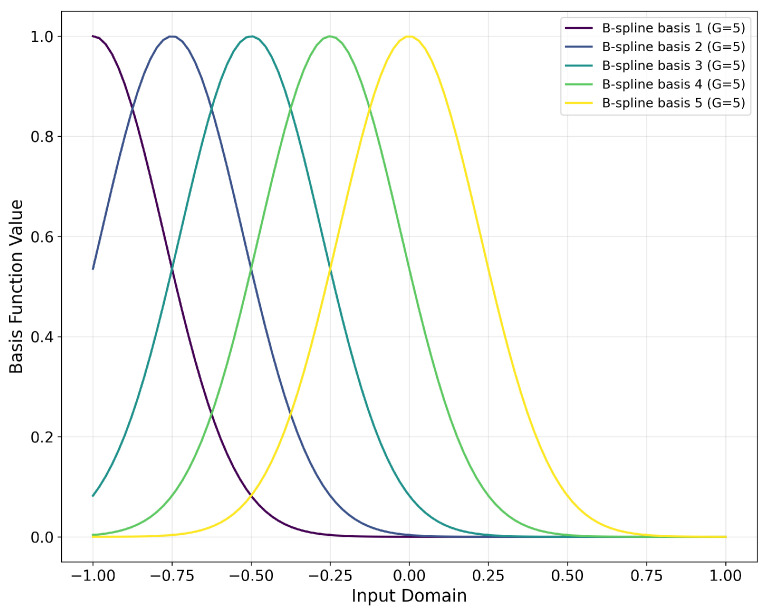
Simplified schematic of B-spline basis function set.

Figure 4 shows a set of simple B-spline function diagrams with five grid points. The definition of the B-spline basis functions relies on the grid partition. We achieve multi-scale analysis through a fixed yet sufficiently dense grid:

(13) $$s_{i}=-1+\frac{2(i-1)}{G-1}$$

For a Lipschitz continuous function $f:{\left[0,\;1\right]}^{d}\to R$, the lower bound of the approximation error for a linear transformation is as follows:

(14) $$\underset{W,b}{\inf}{∥f-\left(W\cdot z+b\right)∥}_{\infty }\geq C\cdot d^{-1/2}$$

where inf denotes taking the infimum (greatest lower bound) over a set, not requiring it to be attained within the set; *f* denotes the clean/ideal ECG signal; ${∥\cdot ∥}_{\infty }$ denotes the maximum absolute error over the entire signal time range, reflecting the worst-case denoising performance; *C* is a constant related to the complexity of the target function, reflecting the complexity of the ECG signal; *d* denotes the input dimension.

This inequality indicates that no matter how carefully the weights W and bias b of the linear model are chosen, the approximation error (worst-case error) is at least $C\cdot d^{-1/2}$. In order to achieve a given small target error ϵ, the required dimension *d* we need is about $O(1/\epsilon^{2})$. That is to say, every time the error is reduced by half, the required dimension we need will increase by about four times. This implies that the error decreases slowly as the dimension *d* increases, making it difficult to effectively capture the nonlinear features and local details in ECG signals.

Based on the Kolmogorov–Arnold representation theorem, the upper bound of the approximation error for the KAN-enhanced transformation is as follows:

(15) $${∥f-\mathrm{KAN}\left(z\right)∥}_{\infty }\leq C^{\prime }\cdot G^{-k}$$

where $C^{\prime }$ is a constant related to the KAN, reflecting the efficiency of the KAN architecture, and is associated with the intrinsic dimensionality of the ECG signal.

This inequality provides an upper bound for the approximation error of the KAN layer, indicating that the error is at most $C^{\prime }\cdot G^{-k}$. This shows the error decreases exponentially as the number of grid points *G* increases, demonstrating that KAN can effectively handle non-stationary features and abrupt changes in ECG signals.

Therefore, we can conclude that KAN, with its excellent theoretical approximation capability, can better learn the complex, nonlinear mapping from noise embeddings to feature transformation parameters, thereby holding promise for achieving superior performance in ECG denoising tasks.

**Figure 5:**
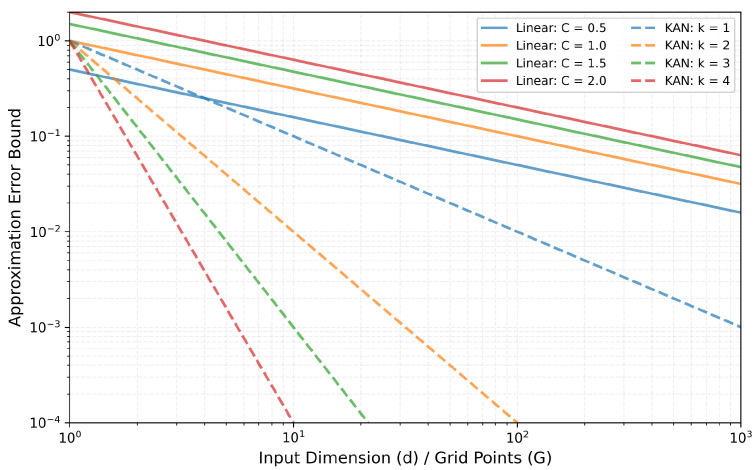
Plot of approximation error for linear transformation and KAN transformation.

From Figure 5 it can be directly observed that the linear transformation has an inherent lower bound which cannot be surpassed, whereas KAN, in theory, can reduce the error indefinitely by increasing the number of grid points *G*. Simultaneously, by adjusting the B-spline order *k*, KAN can balance computational complexity and approximation accuracy. For physiological signals like ECG, which possess characteristic features, higher-order B-splines can better fit abrupt changes such as the QRS complex.

### 3.2. Regularization Methods

In the DeScoD model, regularization is primarily achieved through the inherent stochasticity of the diffusion model and the implicit constraints of the training process. This method relies on the intrinsic properties of the denoising diffusion probabilistic model (DDPM) [27,48], where the iterative process of noise addition and removal itself has a regularizing effect.

Ours introduces a regularization term specifically tailored for KAN (referred to as the KAN regularization loss), which consists of two components: activation sparsity and entropy regularization: activation sparsity regularization and entropy regularization. The loss function can be expressed as follows:

(16) $$\mathrm{Loss}={\mathrm{Loss}}_{\mathrm{main}}+\lambda_{\mathrm{KAN}}\cdot {\mathrm{Loss}}_{\mathrm{KAN}}$$

where $\lambda_{\mathrm{KAN}}$ denotes the importance of the KAN regularization term in the total loss function.

The specific regularization term includes an activation sparsity term and an entropy regularization term:

(17) $${\mathrm{Loss}}_{\mathrm{KAN}}=\gamma_{a}\cdot {\left||W|\right|}_{1}+\gamma_{e}\cdot \left(-\sum p_{i}\log p_{i}\right)$$

where $\gamma_{a}$ denotes the activation penalty coefficient, a hyperparameter greater than 0 controlling the weight of the sparsity penalty in the total regularization loss; ${\left||W|\right|}_{1}$ denotes the $\ell_{1}$ norm of W, encouraging sparsity; $\gamma_{e}$ denotes the entropy penalty coefficient, also a hyperparameter greater than 0 controlling the strength of the entropy penalty; $-\sum p_{i}\log p_{i}$ denotes the classical information entropy.

This regularization term utilizes $\ell_{1}$ norm penalty to reduce model complexity while maximizing entropy to prevent excessive singularity of activation patterns, ensuring that the network can robustly extract and preserve multiple morphological features from noise.

**Figure 6:**
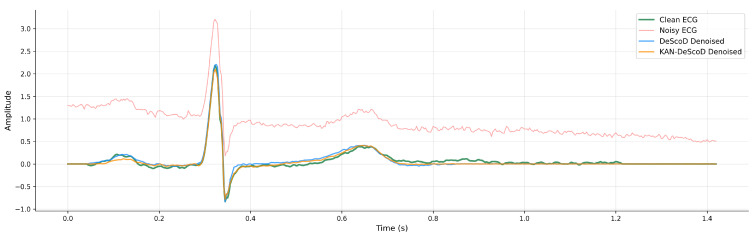
The performance of Kolmogorov–Arnold network enhanced deep score-based diffusion (KAN-DeScoD) and deep score-based diffusion (DeScoD) in dealing with non-stationary noise and baseline wander.

The KAN-DeScoD model introduces a structural prior, and Figure 6 demonstrates that it exhibits superior robustness when handling non-stationary noise and baseline wander, and can improve the identification accuracy of ST-segment and QRS wave group changes. Compared to the DeScoD model, which relies on the implicit regularization of the diffusion process, the explicit regularization in the KAN-DeScoD model provides clearer guidance signals for the optimization process, which can lead to smoother convergence characteristics.

**Figure 7:**
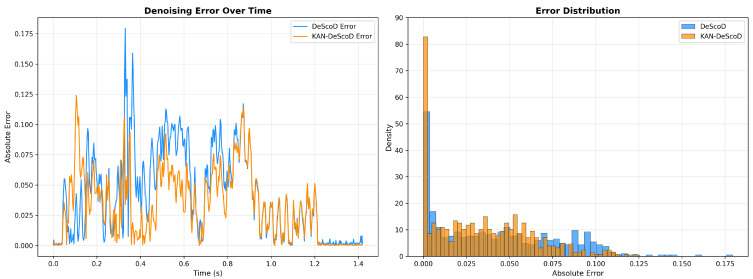
Error analysis of KAN-DeScoD and DeScoD.

Meanwhile, Figure 7 demonstrates that the KAN-DeScoD model exhibits overall lower error compared to the DeScoD model; ours exhibits smaller error oscillation amplitudes near the R peaks, overall lower errors, and a more concentrated error distribution. This indicates that the denoising results of ours are more stable and reliable, with stronger control over key signal points.

## 4. Dataset Description and Performance Metrics

### 4.1. Dataset Description

In this paper, the datasets employed primarily include the QT Database [49] and the MIT-BIH NSTDB [50]. These two databases hold significant importance in the field of ECG signal processing research, providing a high-quality data foundation for model development and evaluation.

The QT Database, a standard database in the field of ECG analysis, provides high-quality, clean heartbeat samples for this paper. In the data splitting strategy, the data were strictly divided into training and test sets based on record names, ensuring that the model’s generalization ability could be evaluated on heartbeats from previously unseen subjects.

The MIT-BIH NSTDB, on the other hand, specifically provides realistic clinical environmental noise, including common noise types such as baseline wander, EMG interference, and motion artifacts. This paper particularly segments the baseline wander noise data from the NSTDB long-term noise recordings into different spatial and temporal segments (for example, using the first half of the first electrode channel and the second half of the second electrode channel as the training and testing sets, respectively), and assigns them separately for the training and testing phases. This ensures that the noise used in the test set has never appeared in the training phase in terms of both space and time. This effectively tests the model’s generalization ability to unknown noise patterns.

Clinical ECG artifacts (baseline wander, EMG interference, etc.) are complex, non-stationary, and spectrally overlapping with the signal. The MIT-BIH NSTDB provides authentic recordings of these noises, making it a gold standard for evaluating denoising robustness in realistic scenarios. Testing against such a database is directly aligned with our paper’s goal of improving model performance and stability under challenging, high-noise conditions, which is of paramount importance for practical clinical application.

The synergistic effect of the two databases is manifested in the synthesis of noisy signals. Through a controlled signal-to-noise ratio (SNR) mechanism, noise fragments from the NSTDB were linearly superimposed onto clean heartbeats from the QT Database to generate the noisy signals used as model input. Specifically, the training phase used combinations of the QT training set and NSTDB training noise fragments, while the testing phase used combinations of the QT test set and NSTDB test noise fragments. This strict data partitioning strategy ensures the objectivity of model evaluation; that is, neither the heartbeat sources nor the noise fragments in the test set were exposed to the model during the training phase, thereby truthfully reflecting the model’s denoising performance on unseen data.

This dataset usage methodology constructs an ideal experimental environment. It preserves the morphological characteristics of real ECG signals and maintains the clinical authenticity of the noise, while also providing a solid foundation for training and reliable evaluation of deep denoising models through precisely controlled SNRs and strict data separation. The final generated dataset comprises four components: noisy training signals and their corresponding clean labels, as well as noisy test signals and their corresponding clean labels, fully supporting an end-to-end supervised learning pipeline.

### 4.2. Evaluation Metrics

To comprehensively evaluate the performance of ECG signal denoising algorithms, this paper selects evaluation metrics from three dimensions: signal fidelity, waveform similarity, and computational efficiency. The *n*-th sample value of the denoised signal is denoted as $\hat{x}\left(n\right)$, the *n*-th sample value of the clean reference signal as x(n), and the number of sampling points in the ECG signal as *N*.

#### 4.2.1. Signal Fidelity Metrics

1. Sum of Squared Distances (SSD): Evaluates the overall distortion degree of the denoised signal.
  (18) $$\mathrm{SSD}=\sum_{n=1}^{N}{\left(x\left(n\right)-\hat{x}\left(n\right)\right)}^{2}$$
2. Maximum Absolute Distance (MAD): Measures the maximum absolute deviation between the denoised signal and the original signal occurring at any single sampling point throughout the entire signal range.
  (19) $$\mathrm{MAD}=\max_{n}\left|x\left(n\right)-\hat{x}\left(n\right)\right|$$
3. Percentage Root-mean-square Difference (PRD): Provides a normalized overall error measurement, evaluating algorithm performance across different datasets.
  (20) $$\mathrm{PRD}=\sqrt{\frac{\sum_{n=1}^{N}{\left(x\left(n\right)-\hat{x}\left(n\right)\right)}^{2}}{\sum_{n=1}^{N}{\left(x\left(n\right)-\mu_{x}\right)}^{2}}}\times 100\%$$

#### 4.2.2. Waveform Similarity Metric

Cosine Similarity (CosSim): Computes the angular similarity between two signal vectors.

(21) $$\mathrm{CosSim}=\frac{\sum_{n=1}^{N}x\left(n\right)\cdot \hat{x}\left(n\right)}{\sqrt{\sum_{n=1}^{N}x{\left(n\right)}^{2}}\cdot \sqrt{\sum_{n=1}^{N}\hat{x}{\left(n\right)}^{2}}}$$

#### 4.2.3. Computational Efficiency Metric

Inference Time: Measures the time required for the algorithm to process a single heartbeat.

(22) $$\mathrm{Time}=\frac{1}{M}\sum_{j=1}^{M}t_{j}$$

where $\max_{n}\left|\cdot \right|$ denotes the maximum absolute value of the signal, i.e., the peak amplitude of the R-wave; *M* denotes the number of test samples; *j* denotes the sample index; $t_{j}$ denotes the processing time for the *j*-th sample.

### 4.3. Experimental Configuration

During the training phase, the DDPM algorithm was used. The number of diffusion steps was set to 1000, the learning rate was fixed at 0.001, $\lambda_{\mathrm{KAN}}$ was set to 0.001, and the Adam optimizer was selected. Table 1 shows the model’s hyperparameters. Training data came from the QT Database and the MIT-BIH NSTDB. ECG signals were segmented into 512-point segments. First, the data was split into training and testing sets based on the record names. Then, the training set was further divided into training and validation sets, not by random sampling of heartbeats, but by splitting the record names into two mutually exclusive sets at a 70%/30% ratio. The loss function combined an $\ell_{1}$ norm reconstruction loss with a KAN regularization term.

During the inference phase, a multi-shot averaging strategy was employed, testing the denoising effect with shot=1, 3, 5, 10. This is based on previous research on the DeScoD model, as well as common practices in the diffusion model field for evaluating multi-sample averaging strategies. Its purpose is to systematically study the convergence trend of model performance as the number of samples increases, and to clearly demonstrate the trade-off between performance improvement and increased computation time. Shot = 1 is used as the baseline, while 3, 5, and 10 represent typical values for gradually increasing sampling to observe stability. This strategy reduces errors introduced by randomness by performing multiple independent samplings and taking the average. The noise level was divided into four amplitude intervals: 0.2–0.6, 0.6–1.0, 1.0–1.5, and 1.5–2.0, to evaluate the model’s robustness under different noise intensities.

The hardware environment was configured with an Ubuntu 22.04 system, equipped with a single RTX 5090 GPU (32 GB VRAM) and an Intel Xeon Platinum 8470Q CPU (25 vCPUs). Software dependencies included PyTorch 2.7.0, Python 3.12, and CUDA 12.8, ensuring computational efficiency and compatibility. The experimental code was based on a modular design, supporting automated training-validation-testing pipelines, with hyperparameters managed through a YAML file.

## 5. Experimental Results

Since the superiority of the DeScoD model architecture over other classic ECG denoising models has been well established [32], this paper directly and specifically compares the KAN-DeScoD model with the original DeScoD model. The experimental results demonstrate that the KAN-DeScoD model achieved significant and stable performance enhancement in the ECG signal denoising task. The evaluation results are primarily presented from two dimensions: overall performance across different inference shots and model robustness under different noise levels. Meanwhile, all performance metrics reported in this section (including Table 2 and Table 3) were calculated on a completely independent test set strictly divided according to the subject separation principle.

### 5.1. Performance Under Different Inference Shots

From Table 2, it can be seen that in terms of overall performance, the KAN-DeScoD model demonstrates advantages across different inference shots. When the number of inference shots increases from 1 to 10, key metrics measuring signal distortion, such as SSD, MAD, and PRD, show a decrease, while CosSim, representing signal morphological fidelity, consistently rises. This indicates that through multi-shot averaging, the stability and accuracy of the model’s predictions are systematically improved. It is noteworthy that the advantage of the KAN model over the original model persists across all metrics as the sampling count increases. For example, with 10 inference shots, PRD decreased from 38.364% to 37.747%, a noticeable reduction. The cost of this performance improvement is increased computation time. Due to its higher structural complexity, the KAN model’s inference time is approximately 30–35% longer than that of the original model. However, considering its significant benefits in signal fidelity, this cost is reasonable and acceptable.

### 5.2. Robustness Under Different Noise Levels

As can be seen from Table 3, in terms of noise robustness, the KAN-DeScoD model demonstrates stronger stability across different noise intensity intervals. Across the four defined noise levels, from the relatively low 0.2–0.6 to the highest 1.5–2.0, all distortion metrics of ours are superior to those of the original model, and its CosSim is higher. Particularly noteworthy is that the advantage of ours becomes more prominent in high-noise intervals. For instance, in the highest noise level interval of 1.5–2.0, the PRD significantly decreased from 51.556% to 50.042%, representing a relative improvement of approximately 3.0%. This strongly proves that the KAN endows the model with stronger feature extraction and nonlinear fitting capabilities, enabling it to effectively restore the key morphological features of the ECG signal even under severe noise contamination. In summary, the introduction of KAN not only generally improves the model’s denoising accuracy but, more importantly, enhances the model’s robustness when dealing with high-intensity noise. This holds significant importance for addressing complex noise environments in practical clinical applications.

## 6. Discussion

This experiment explored the effect of introducing the KAN into the DeScoD model to enhance ECG signal denoising performance. The experimental results confirm the effectiveness of this improvement.

From the perspective of performance improvement, the KAN indeed brought positive enhancements to the model. The performance comparison across different inference shots presented in Table 2 shows that for the four core metrics, SSD, MAD, PRD, and CosSim, ours consistently outperformed the DeScoD model at each sampling count. This advantage remained stable as the number of shots increased. These improvements benefit from the smooth and adaptive nonlinear transformation capabilities provided by the B-spline basis functions used in KAN. Compared to the fixed activation functions of traditional MLP, KAN can more flexibly learn and approximate complex functional relationships, thereby better preserving the inherent, clinically relevant morphological features of ECG signals during the denoising process.

However, the experimental data also clearly indicate that reduced computational efficiency is a significant issue. In Table 2, the KAN-DeScoD model shows an increase in inference time with the same number of samples. The core value of introducing KAN lies in breaking the physical limitations imposed by the theoretical error lower bound faced by traditional linear transformations when approximating non-stationary ECG features, as well as enhancing robustness in denoising under non-stationary noise. Although there is currently a trade-off in inference speed, it provides a novel backbone network with infinite approximation potential for generating denoising models. At the same time, KAN inherently offers interpretability and parameter efficiency, and by exploring lightweight variants [51,52], it is expected to improve computational efficiency.

The test results under different noise levels in Table 3 strongly demonstrate the model’s robustness. From the low-noise interval (0.2–0.6) to the high-noise interval (1.5–2.0), the KAN-DeScoD model consistently outperformed the original model on almost all metrics. Particularly in the most challenging high-noise level (1.5–2.0), ours achieved a PRD of 50.042%, approximately 1.5 percentage points lower (a relative improvement of about 3.0%) than the original model’s 51.556%, and SSD also decreased from 5.052 mV to 4.996 mV. This indicates that the KAN-DeScoD model possesses greater robustness in signal recovery when dealing with high-intensity noise contamination. Compared to some traditional filters that are effective only for specific noise types or intensities, ours demonstrates broader adaptability.

## 7. Limitations and Future Work

Despite the outstanding denoising performance demonstrated by the KAN-DeScoD model, there are still limitations. Due to the introduction of the KAN layer, ours exhibits increased inference time under the same number of samples. This increase in time overhead directly stems from the more complex computational process of KAN compared to the standard linear layer, especially the calculation of B-spline basis functions and parameter optimization. Future optimizations can be explored from the following aspects:

1. Explore other lightweight KAN variants [51,52], as well as methods such as dynamic mesh updating and model pruning to reduce the computational complexity of B-splines.
2. Further improve the parameter selection process by introducing self-optimization strategies [53].

This paper primarily validates the model on standardized datasets. However, its practical application in real-world scenarios still requires further investigation. Future research can focus on integrating the model into low-power embedded systems to achieve real-time cardiac monitoring, as well as evaluating the impact of denoising on downstream clinical tasks (such as automatic arrhythmia detection or ST segment analysis) to ensure that the KAN-enhanced nonlinear mapping preserves all key diagnostic markers. Moreover, the current research focuses on single-lead ECG signals, and its scalability in terms of parameter efficiency and spatiotemporal relationship modeling when processing multi-lead signals requires further validation.

## 8. Conclusions

In summary, integrating KAN into the DeScoD model for the first time is a beneficial attempt. While maintaining the powerful generative capabilities of diffusion models, it enhances denoising accuracy and signal fidelity through a more powerful function approximator, with advantages particularly evident when processing high-noise signals. This improvement provides a new perspective for the field of ECG signal processing. However, the associated increase in computational cost is a practical factor that must be considered for real-world deployment. Future work needs to find a better balance between improving performance and ensuring efficiency, and to comprehensively evaluate its clinical utility through testing on broader and more complex datasets.

## Figures and Tables

**Table 1:** Key Hyperparameters of the Kan-DeScoD Model.

Category	Parameter	Value
KAN Architecture	Grid Size (*G*)	5
	Spline Order (*k*)	3
	Base Activation Function	SiLU
	Grid Range	[−1, 1]
	Scale Base/Spline	1.0/1.0
	Standalone Scale Spline	Enabled
Diffusion (DDPM)	Diffusion Steps (*T*)	50
	Noise Schedule	Quadratic
	Beta Start	0.0001
	Beta End	0.5
	Conditioning Strategy	Signal-based
Training Settings	Hidden Features	80
	Total Epochs	200
	Batch Size	96
	Initial Learning Rate	$1.0\times 10^{-3}$
	LR Scheduler	γ=0.1,step=150

**Table 2:** Performance comparison for different shots.

Model	Shot	SSD (mV^2^)	MAD (mV)	PRD (%)	CosSim (a.u.)	Time (s)
DeScoD	1	4.650	0.355	43.204	0.908	60
	3	3.692	0.321	39.791	0.927	177
	5	3.465	0.313	38.959	0.931	292
	10	3.320	0.308	38.364	0.934	605
KAN-DeScoD	1	4.558	0.356	42.438	0.911	81
	3	3.574	0.320	39.132	0.929	241
	5	3.410	0.312	38.428	0.933	401
	10	3.263	0.306	37.747	0.936	795

**Table 3:** Performance comparison for different noise ranges (10 shots).

Model	Noise	SSD (mV^2^)	MAD (mV)	PRD (%)	CosSim (a.u.)
DeScoD	0.2–0.6	1.506	0.187	24.523	0.971
	0.6–1.0	2.557	0.264	32.587	0.952
	1.0–1.5	3.626	0.335	40.899	0.929
	1.5–2.0	5.052	0.411	51.556	0.896
KAN-DeScoD	0.2–0.6	1.489	0.187	24.361	0.972
	0.6–1.0	2.511	0.264	32.482	0.954
	1.0–1.5	3.532	0.333	40.400	0.931
	1.5–2.0	4.996	0.410	50.042	0.899

## Data Availability

The data presented in this paper are openly available in PhysioNet at https://physionet.org/content/qtdb/ (accessed on 1 November 2025) and https://physionet.org/content/nstdb/ (accessed on 1 November 2025).

## Abbreviations

The following abbreviations are used in this manuscript:

DeScoD	Deep score-based diffusion
ECG	Electrocardiogram
KAN	Kolmogorov–Arnold network
KAN-DeScoD	Kolmogorov–Arnold network enhanced deep score-based diffusion
NSTDB	Noise Stress Test Database
EMG	Electromyographic
GAN	Generative adversarial network
MLP	Multi-layer perceptrons
DDPM	Denoising diffusion probabilistic model
SNR	Signal-to-noise ratio
SSD	Sum of squared distances
MAD	Maximum absolute distance
PRD	Percentage root-mean-square difference
CosSim	Cosine similarity
